# Incidence and risk factors of infertility among couples who desire a first and second child in Shanghai, China: a facility-based prospective cohort study

**DOI:** 10.1186/s12978-022-01459-x

**Published:** 2022-07-08

**Authors:** Chenfeng Zhu, Li Yan, Chuqing He, Yang Wang, Jiahao Wu, Luting Chen, Jian Zhang

**Affiliations:** 1 Department of Obstetrics and Gynecology, International Peace Maternity and Child Health Hospital, School of Medicine, Shanghai Jiaotong University, Shanghai, China; 2 Pre-Pregnancy center, International Peace Maternity and Child Health Hospital, School of Medicine, Shanghai Jiaotong University, Shanghai, China; 3 Shanghai Key Laboratory Embryo Original Diseases, Shanghai, China; 4 Shanghai Municipal Key Clinical Specialty, Shanghai, China

**Keywords:** Second child intention, First child intention, Epidemiology, Incidence, Infertility, Risk factor

## Abstract

**Background:**

With the implementation of the two-child policy in China, more couples have expressed the desire to have another child. We conducted this study to evaluate the incidence of infertility and risk factors in couples intending to have a first and second child.

**Methods:**

From 2013 to 2017, a prospective cohort study was conducted at the pre-pregnancy center of the International Peace Maternal and Child Health Hospital. The participants were selected by screening and random sampling couples who came to the pre-pregnancy center. Data regarding patient sociodemographic characteristics, reproductive and gynecological history, male disease history, and laboratory and imaging examination results were collected. Couples were followed up every 3 months until pregnancy or for 12 months, whichever came first. Multi-factor logistic regression was used to analyze risk factors for infertility. Adjusted odds ratios (aORs) and corresponding 95% confidence intervals (CIs) were calculated and adjusted for potential confounding factors.

**Results:**

The overall infertility incidence was 16.95% (369/2177). The infertility incidence of “first child intention” and “second child intention” was 19.30% (355/1839) and 4.14% (14/338), respectively. This study found great differences in both infertility rate (P < 0.001) and risk factors between the two groups. Risk factors for “first child intention” infertility included advanced age (> 35 years) (aOR = 1.70, 95% CI 1.27–2.28), abnormal body mass index (BMI) (aOR = 1.58, 95% CI 1.31–6.26), longer menstrual periods (aOR = 4.47, 95% CI 2.25–8.88), endometrial polyps (aOR = 2.52, 95% CI 1.28–4.97), polycystic ovarian syndrome (PCOS) (aOR = 6.72, 95% CI 1.79–7.39), salpingostomy (aOR = 3.44, 95% CI 1.68–7.07), and history of mycoplasma (aOR = 1.54, 95% CI 1.09–2.40). However, in the “second child intention” group, clinical risk factors slightly differed and included leiomyoma (aOR = 5.60, 95% CI 1.06–29.76), and higher age (> 40 years) (aOR = 7.36, 95% CI 1.01–53.84).

**Conclusion:**

The overall infertility rate in Shanghai is similar to that of other large cities in China. Marriage at advanced ages has become increasingly common. As such, the government must consider subsidies to encourage childbirth at childbearing ages, which can improve fertility levels.

**Supplementary Information:**

The online version contains supplementary material available at 10.1186/s12978-022-01459-x.

## Plain Language Summary

Infertility is defined as pregnancy failure after at least 12 months of regular unprotected sexual intercourse. Few researchers have investigated the infertility rate in Shanghai in the past 15 years, and little attention has been paid to the infertility of couples hoping to have a second child. We conducted a prospective cohort study in Shanghai to evaluate infertility incidence and risk factors in couples intending to have a first or second child. The investigators administered a questionnaire survey to the participants and followed them for 1 year. Finally, 1839 couples intending to have a first child and 338 couples intending to have a second child were included in this study. The overall infertility incidence was 16.95% (369/2177). However, the infertility incidence of the “first child intention” and “second child intention” groups was 19.30% (355/1839) and 4.14% (14/338), respectively. Risk factors for “first child intention” infertility included advanced age (> 35 years), abnormal body mass index (BMI), longer menstrual periods, endometrial polyps, polycystic ovarian syndrome (PCOS), salpingostomy, and history of mycoplasma; in the “second child intention” group, clinical risk factors slightly differed and included leiomyoma and advanced age (> 40 years). Since studies have shown large differences in infertility risk factors between the two groups, early and targeted intervention for couples in different high-risk groups can help reduce infertility.

**Supplementary Information:**

The online version contains supplementary material available at 10.1186/s12978-022-01459-x.

## Background

Infertility is a common medical problem, though its influencing factors have not been elucidated [1]. However, previous studies have found that diseases of the reproductive system and social and psychological factors contribute to infertility [2, 3]. Additionally, due to differences in region, environment, and economy, infertility incidence significantly varies around the world [4, 5].

Previous studies have found that 12-month infertility rates were 15.6% (2009–2010) and 10.5% (2007) in Canada and Scotland, respectively [6, 7]. The overall prevalence of infertility in Iran was 8% in 2006 [8]. A 2007 survey in China involving 17,275 women from 8 provinces estimated that the overall infertility rate was 15.6% [9], while the infertility rate in Shanghai was 9% in 2002 [10]. However, these results were based on data that are now outdated, and the current infertility rate in Shanghai is unknown.

Infertility is a national concern that causes emotional, financial, and societal burdens [11]. Surveys have shown that the financial burden of infertility in China in 2008 was approximately 11.4 billion to 32.5 billion dollars [12]. Infertility rates have continued to increase in the Chinese population, from 6.7% in 2005 to 15.5% in 2018 [9]. As the incidence of infertility increases, so does its societal burden. By the end of 2018, 497 assisted reproductive technology (ART) centers were approved by the Chinese government; the total number of human ART cycles per year has exceeded 1 million, and the number of babies born has exceeded 300,000 [13]. Therefore, infertility is a problem that requires urgent attention.

According to the World Health Organization (WHO) definition, infertility is the failure to become pregnant after at least 12 months of regular unprotected sexual intercourse [14]. Primary infertility is defined as infertile couples with no pregnancy history, while secondary infertility is defined as infertile couples with a history of pregnancy [15]. Compared with primigravida, increasing age and other potential factors can compromise the fertility of women who intend to have a second child. Recent research has focused on the prevalence and risk factors for primary and secondary infertility [16], though too little attention has been paid to the infertility of couples intending to have a second child.

Since China implemented the second-child policy, approximately 90 million women have been allowed to have a second child [17]. Moreover, recent evidence has demonstrated that of these 90 million women, approximately 60% are over 35, and 50% are over 40 [18]. Therefore, we performed a prospective cohort study in Shanghai, based on a few researchers investigating infertility incidence in the past 15 years to evaluate the incidence and risk factors of infertility in couples intending to have a first or second child.

## Materials and methods

### Study design and participants

We performed a single-center, prospective cohort study in pre-pregnancy centers in Shanghai, China, from January 2013 to December 2017. Since 2012, China has performed pre-pregnancy check-ups, prompting couples trying to become pregnant to seek services from these institutions [19]. These pre-pregnancy centers provide pre-pregnancy education, consultation, and ordinary examinations (including infectious diseases and reproductive system examination for women) for couples of childbearing age in surrounding communities.

The inclusion criteria included: (1) aged 20 to 49 years; (2) couples who intend to become pregnant and engage in regular sexual activity over the following year. The exclusion criteria included: (1) BMI < 17 or > 33 kg/m^2^); (2) couples who did not plan to become pregnant or had a history of infertility; (3) couples whose laboratory findings did not allow them to be pregnant within the next year or those who planned to have a third child or more. Written informed consent was obtained from all participants.

### Procedures

Based on the inclusion and exclusion criteria, proportionate sampling was used to select the study participants, who were recruited couples of childbearing ages who came to the pre-pregnancy center from January 2013 to December 2017. Participants were recruited by random sampling in an equal ratio (15:1) from the eligible population based on the order they came to the hospital. After informed consent was obtained, information was collected from each participant by a trained investigator, including sociodemographic characteristics (e.g., age, marital status, education, occupation, individual annual income, smoking status), history of reproduction, and gynecology (e.g., number of pregnancies, pregnancy outcomes, age at menarche, menstrual cycle, menstrual duration, menstrual blood volume, medical history, operative history), disease history of the male (e.g., medical history, operative history), and pre-pregnancy medical examination results (e.g., serological antibody, pelvic ultrasound).

Follow-ups to assess pregnancy outcomes were evaluated by telephone or face-to-face interviews. Follow-ups were performed by a well-trained investigator via telephone every 3 months until delivery or for 12 months, whichever came first. Follow-up questionnaires included: Are you currently pregnant? When was the last menstrual period before pregnancy? How long have you been pregnant, and have you identified any new gynecological disorders?

Our study defined infertility as not becoming pregnant through regular sex without contraception for at least 12 months. Based on whether participants were infertile, couples intending to have a first child were then classified into the “infertility of first child intention” group and the “fertility of first child intention” group. During the same study period, couples intending to have a second child were classified into the “infertility of second child intention” group and the “fertility of second child intention” group. According to the definition of infertility, the infertility incidence was calculated as the number of infertile women divided by the number of women intending to become pregnant.

### Statistical analysis

Based on a previous pilot study and relevant studies [9, 10], we assumed that the infertility rate among couples was 15%. With an error margin of 2% and a two-sided 95% confidence interval (CI), this required total sample size of 1273. To minimize the sampling error, we calculated a final sample size that was 1.5-fold the previous one, resulting in a total sample size of 2000.

Infertility was considered a binary outcome, and corresponding 95% CIs were calculated assuming a binomial distribution of the observed number of events. Univariable conditional logistic regression analysis was also used to calculate crude odds ratios (ORs) and 95% CIs. Multivariable logistic regression analysis was performed to explore potential risk factors and corresponding ORs. Before constructing the logistic regression model, a multicollinearity analysis was performed between independent variables included in the regression models. We chose the covariates examined by multivariable logistic regression based on their clinical relevance to infertility and the results of univariate analysis. Forward stepwise regression was used to combine factors related to infertility (female age and BMI, male BMI, menstrual duration, female medical history of leiomyoma, endometrial polyp, PCOS, endometriosis, salpingostomy, and chlamydia genitalium) in a multivariate regression model [20]. Statistical analysis was performed using IBM SPSS Version 21.0 (IBM Corp., Armonk, NY, USA). All p-values were estimated using two-sided tests, and differences were considered statistically significant when p < 0.05.

## Results

**Fig. 1:**
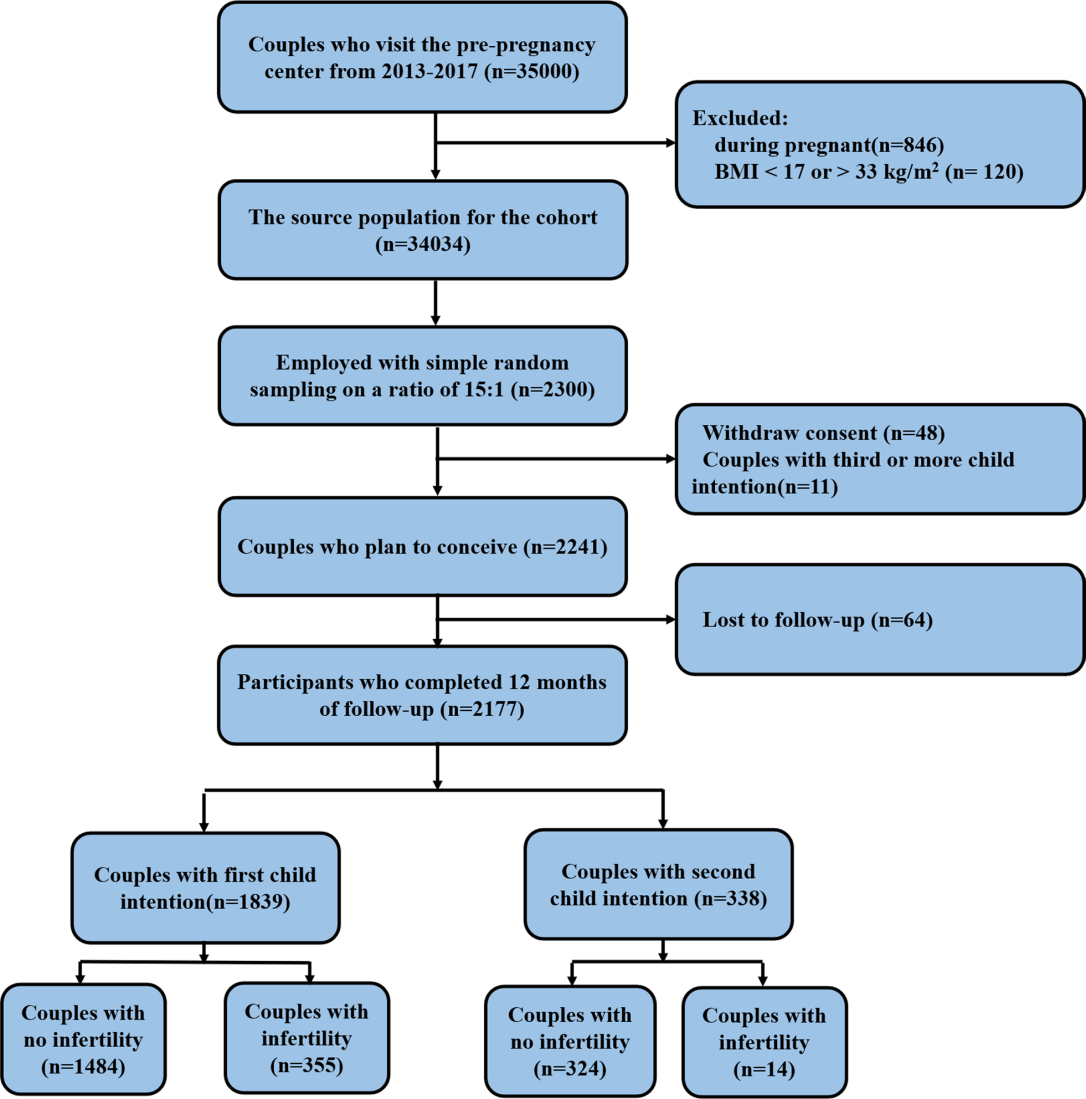
Recruitment profile of the study

From January 2013 to December 2017, 35,000 couples visited the pre-pregnancy center. After excluding 846 couples who were pregnant and 120 couples with extreme BMI values, 2300 participants in the cohort were selected from 34,034 couples at a 15:1 ratio. However, 48 (2.09%) couples who were worried about disclosing their private information refused to participate in the investigation, 64 (2.78%) were lost during the follow-up period, and 11 (0.50%) couples already had two or more children. Finally, 1839 couples intending to have a first child and 338 couples intending to have a second child were included in this study (recruitment flow chart is shown in Fig. 1).

### Baseline characteristics

Table 1 shows the differences in the sociodemographic and socioeconomic characteristics of couples in this study. The mean (± standard deviation [SD]) age was 29.76 (± 3.71) years for all women and 31.36 (± 4.39) years for all men. Among couples with “first child intention,” the mean (± SD) age was 29.50 (± 3.52) for women and 31.12 (± 4.22) for men. Among couples with “second child intention,” the female and male mean (± SD) ages were 31.11 (± 4.36) and 32.53 (± 4.97), respectively. Furthermore, the mean BMI (± SD) values for all women and men were 21.08 (± 2.69) kg/m^2^ and 23.67 (± 2.82) kg/m^2^, respectively. Among couples with “first child intention,” the mean BMI (± SD) was 21.07 (± 2.70) kg/m^2^ for women and 23.71 (± 2.87) kg/m^2^ for men. Among couples with “second child intention,” the mean BMI (± SD) was 21.15 (± 2.60) kg/m^2^ for women and 23.52 (± 2.59) kg/m^2^ for men.

**Table 1:** Sociodemographic characteristics of study participants

	All couples				P value ^d^	Fertility (N = 324)	Infertility^a^ (N = 14)	P value^d^
	N = 2177		Fertility (N = 1484)	Infertility^a^ (N = 355)				
	n^b^	%	n^b^ (%)	n^b^(%)		n^b^(%)	n^b^(%)	
Age, years, women								
20–24	133	6.11	92 (6.23)	19 (5.35)	< 0.001	21 (6.50)	1 (7.14)	0.038
25–29	1096	50.34	818 (55.38)	153 (43.10)		112 (34.67)	4 (28.57)	
30–34	743	34.13	475 (32.16)	137 (38.59)		124 (38.39)	4 (28.57)	
35–39	176	8.08	84 (5.69)	38 (10.70)		58 (17.96)	3 (21.43)	
≥ 40	29	1.33	8 (0.54)	8 (2.25)		8 (2.48)	2 (14.29)	
Age, years, men								
20–24	55	2.79	38 (2.96)	6 (1.99)	0.002	9 (3.02)	1 (8.33)	0.631
25–29	715	36.31	526 (41.03)	97 (32.12)		88 (29.53)	2 (16.67)	
30–34	782	39.72	533 (41.58)	126 (41.72)		111 (37.25)	5 (41.67)	
35–39	328	16.66	142 (11.15)	55 (18.21)		62 (20.81)	2 (16.67)	
≥ 40	89	4.52	42 (3.28)	18 (5.96)		27 (0.34)	2 (16.67)	
BMI^c^ kg/m^2^, women								
< 18.5	37	2.03	180 (14.42)	35 (11.36)	< 0.001	40 (13.70)	2 (15.38)	0.905
18.5–23.9	1022	56.18	939 (75.24)	202 (65.58)		213 (72.95)	10 (76.92)	
24–26.9	545	29.96	105 (8.41)	47 (15.26)		29 (9.93)	1 (7.69)	
≥ 27	215	11.82	24 (1.92)	24 (7.79)		10 (3.42)	0	
BMI^c^ kg/m^2^, men								
< 18.5	37	2.03	24 (2.00)	11 (3.65)	0.008	2 (0.69)	0	0.792
18.5–23.9	1022	56.18	691 (57.44)	142 (47.18)		175 (60.14)	8 (66.67)	
24–26.9	545	29.96	342 (28.43)	101 (33.55)		93 (31.96)	4 (33.33)	
≥ 27	215	11.82	146 (12.14)	47 (15.61)		21 (7.22)	0	
Occupation, women								
Employed	1768	90.95	1210 (92.30)	284 (91.91)	0.950	256 (85.62)	11 (84.62)	0.343
Self-employed	84	4.32	46 (3.51)	12 (3.88)		21 (7.02)	2 (15.38)	
Unemployed	92	4.73	55 (4.20)	13 (4.21)		22 (7.36)	0	
Education attainment, women								
High school or low	119	6.08	65 (4.95)	26 (8.33)	0.039	24 (7.82)	4 (30.77)	0.016
Junior college or university	1534	78.39	1029 (78.37)	243 (77.88)		244 (79.48)	8 (61.54)	
Graduate or above	304	15.53	219 (16.68)	43 (13.78)		39 (12.70)	1 (7.69)	
Education attainment, men								
High school or low	110	5.69	58 (4.46)	20 (6.60)	0.106	28 (9.157)	4 (30.77)	0.030
Junior college or university	1471	76.10	1002 (77.14)	239 (78.88)		214 (69.93)	8 (61.54)	
Graduate or above	352	18.21	239 (18.40)	44 (14.52)		64 (20.92)	1 (7.69)	
Average monthly incomes (¥) of each couple								
< 10,000	117	6.35	61 (4.94)	27 (9.41)	0.012	27 (9.15)	1 (7.69)	0.903
10,000–20,000	424	23.02	299 (24.21)	62 (21.60)		58 (19.66)	2 (15.38)	
> 20,000	1301	70.63	875 (70.85)	198 (68.99)		210 (71.19)	10 (76.92)	

^a^Faliure to conceive after regular unprotected sexual intercourse for 1 year

^b^The sum does not necessarily equal the sample size for all variables because of missing data

^c^BMI is defined as Body mass index; Body mass index is defined as weight in kilograms divided by the square of height in meters

^d^Pearson’s χ^2^ test

Most couples completed had a junior college, university, or high level of education. More than 90% of the women were employed, and most households had annual incomes exceeding 20,000 yuan.

In the “couples with first child intention” group, there were more couples that were older, had a higher BMI, and had lower incomes in the infertility group than in the fertility group. Furthermore, women in this group with lower education were more likely to be infertile. In the “couples with second child intention” group, the infertility group had older, lower-educated women and lower-educated men.

### The incidence of infertility

Among all couples who planned to become pregnant (n = 2177, the overall incidence of infertility was 16.95% (95% CI 15.37–18.53%; infertility = 369, fertility = 1803). The incidence of “infertility of first child intention” was 19.30% (95% CI 17.50–21.11%; infertility = 355, fertility = 1484). In contrast, the incidence of “infertility of second child intention” was 4.14% (95% CI 2.01–6.28%; infertility = 14, fertility = 324).

**Fig. 2:**
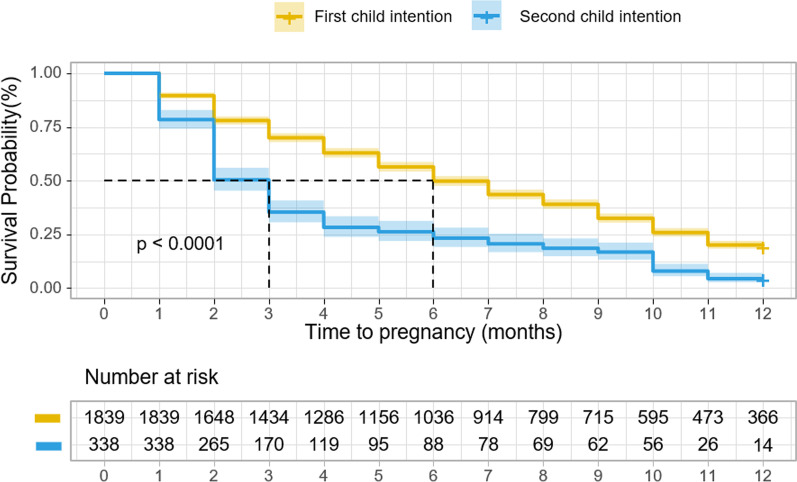
Kaplan–Meier curve for time to pregnancy. Kaplan–Meier curve for time to pregnancy according to child intention. Quicker to get pregnant is indicated for women with second child intention (log-rank P < 0.001)

The infertility rate between the two groups was statistically different (P < 0.001). Figure 2 shows Kaplan–Meier curves of time to pregnancy between the “first child intention” group and “second child intention” group. The “second child intention” group became pregnant faster and had a lower infertility rate than the “first child intention” group (log-rank P < 0.001).

**Fig. 3:**
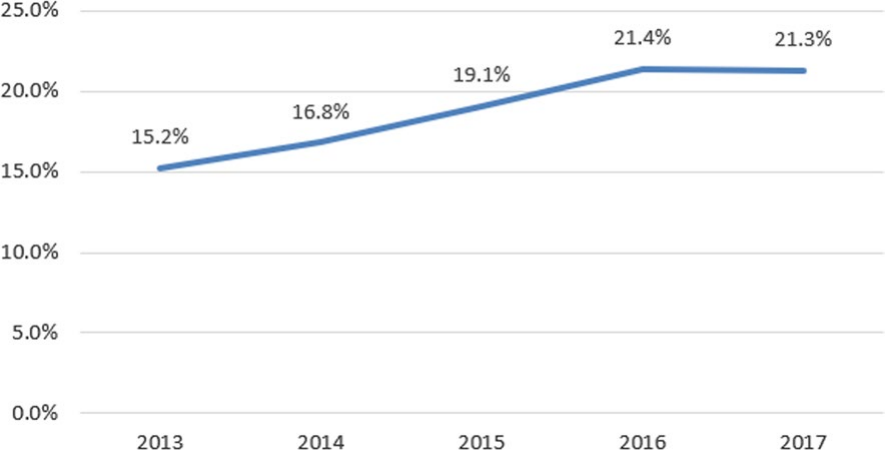
Infertility rates in different years (2013: 15.2%; 2014: 16.8%; 2015: 19.1%; 2016: 21.4%; 2017: 21.3%) (Trend P test < 0.01)

We also calculated annual infertility rates based on the year participants were enrolled and found that the infertility rate increased each year (Fig. 3, 2013: 15.2%; 2014: 16.8%; 2015: 19.1%; 2016: 21.4%; 2017: 21.3%). (Trend P test < 0.01).

### Univariate analysis

Table 2 shows the crude unadjusted ORs and their 95% CIs for the relationship between infertility and female medical history. In the “couples with first child intention” group, women with a longer menstrual cycle and longer menstrual periods were more likely to be infertile compared with women who had normal menstrual periods. Regarding the medical history, infertility was associated with factors such as leiomyoma, ovarian cysts, endometrial polyps, endometriosis, polycystic ovarian syndrome (PCOS), and a history of lower genital tract infections (mycoplasma, chlamydia, and condyloma acuminate). Moreover, among infertile couples, many women had a history of surgery such as uterine myomectomy, salpingostomy, transcervical polyp resection, and hysteroscopic adhesiolysis, which were associated with a significantly higher risk of infertility. In the “couples with second child intention” group, the crude OR of infertility in women with later menarche and history of leiomyoma was significantly lower than that of women who did not.

**Table 2:** Menstrual and medical history of women

	All couples		P value^c^	First child intention		P value^c^	Second child intention		P value^c^
	Infertility^a^ (N = 369)	Fertility (N = 1808)		Infertility^a^ (N = 355)	Fertility (N = 1484)		Infertility^a^ (N = 14)	Fertility (N = 324)	
	n^b^ (%)	n^b^ (%)		n^b^ (%)	n^b^ (%)		n^b^ (%)	n^b^ (%)	
Menstrual history									
Menarche (year)									
< 13	49 (14.63)	225 (13.71)	0.101	46 (14.56)	171 (12.85)	0.319	2 (14.29)	53 (17.32)	< 0.001
13–14	245 (73.13)	1273 (77.57)		234 (74.05)	1037 (12.85)		7 (50.00)	234 (76.47)	
> 14	41 (12.23)	143 (8.71)		36 (11.39)	123 (9.24)		5 (35.71)	19 (6.21)	
Menstrual cycle (days)									
21–35	278 (81.52)	1556 (93.29)	< 0.001	259 (80.43)	289 (92.99)	< 0.001	14 (100.00)	289 (94.44)	1.000
> 35	63 (18.48)	112 (6.72)		63 (19.57)	95 (7.01)		0 (0)	17 (5.56)	
Menstrual duration (days)									
2–7	317 (92.69)	1629 (97.60)	< 0.001	299 (92.57)	1328 (98.23)	< 0.001	13 (92.86)	294 (95.77)	0.471
> 7	25 (7.31)	40 (2.40)		24 (7.43)	24 (1.77)		1 (7.14)	13 (4.23)	
Dysmenorrhea									
None	148 (43.529)	728 (43.776)	0.936	139 (43.30)	568 (42.14)	0.894	8 (57.14)	157 (50.97)	0.903
Occasional	107 (31.471)	508 (30.547)		102 (31.78)	428 (31.75)		3 (21.43)	76 (24.68)	
Regular	85 (25.000)	427 (25.676)		80 (24.92)	352 (26.11)		3 (21.43)	75 (24.35)	
Gynecological history									
Pelvic inflammation^d^									
No	320 (93.84)	1590 (95.21)	0.292	303 (93.81)	1292 (95.56)	0.191	12 (92.31)	293 (94.21)	0.551
Yes	21 (6.16)	80 (4.79)		20 (6.19)	60 (4.44)		1 (7.69)	18 (5.79)	
Adenomyosis^d^									
No	340 (97.98)	1671 (99.10)	0.047	335 (97.95)	1426 (99.03)	0.157	14 (100)	318 (100)	NA
Yes	7 (2.02)	14 (0.83)		7 (2.05)	14 (0.97)		0 (0)	0 (0)	
Leiomyoma^d^									
No	301 (83.15)	1607 (90.84)	< 0.001	287 (83.67)	1301 (90.16)	0.001	10 (71.43)	299 (93.73)	0.013
Yes	61 (16.85)	162 (9.16)		56 (16.33)	142 (9.84)		4 (28.57)	20 (6.27)	
Ovarian cyst^d^									
No	308 (85.32)	1624 (91.96)	< 0.001	293 (85.67)	1328 (92.16)	< 0.001	13 (92.86)	291 (91.51)	1.000
Yes	53 (14.68)	142 (8.04)		49 (14.33)	113 (7.84)		1 (7.14)	27 (8.49)	
Endometrial polyp^d^									
No	309 (90.62)	1616 (97.12)	< 0.001	291 (90.37)	1307 (97.10)	< 0.001	13 (92.86)	304 (97.75)	0.3
Yes	32 (9.38)	48 (2.89)		31 (9.63)	39 (2.90)		1 (7.14)	7 (2.25)	
Endometriosis^d^									
No	320 (93.57)	1637 (98.08)	< 0.001	304 (94.12)	1320 (97.71)	0.002	13 (92.86)	310 (99.68)	0.084
Yes	22 (6.43)	32 (1.92)		19 (5.88)	31 (2.29)		1 (7.14)	1 (0.32)	
PCOS^d^									
No	311 (86.15)	1721 (97.51)	< 0.001	292 (85.38)	1407 (97.71)	< 0.001	14 (100)	307 (96.54)	1.000
Yes	50 (13.85)	44 (2.49)		50 (14.62)	33 (2.29)		0 (0)	11 (3.46)	
Surgical history									
Uterine myomectomy^e^									
No	335 (96.54)	1670 (99.05)	0.001	317 (96.65)	1352 (98.98)	0.004	14 (100)	311 (99.36)	1.000
Yes	12 (3.46)	16 (0.95)		11 (3.35)	14 (1.02)		0 (0)	2 (0.64)	
Salpingostomy^e^									
No	323 (93.08)	1663 (98.64)	< 0.001	13 (92.86)	1347 (98.61)	< 0.001	13 (92.86)	309(98.72)	0.198
Yes	24 (6.92)	23 (1.36)		22 (6.71)	19 (1.39)		1 (7.14)	4(1.28)	
Endometrial polypectomy^e^									
No	333 (95.97)	1666 (98.81)	< 0.001	315 (96.04)	1349 (98.76)	0.004	14 (100)	309 (98.72)	1.000
Yes	14 (4.04)	20 (1.19)		13 (3.96)	17 (1.24)		0 (0)	4 (1.28)	
Hysteroscopic adhesiolysis^e^									
No	339 (97.70)	1673 (99.23)	0.037	320 (97.56)	1354 (99.12)	0.039	14 (100)	312 (99.68)	NA
Yes	8 (2.31)	13 (0.77)		8 (2.44)	12 (0.88)		0 (0)	1 (0.32)	
Genital tract infection history									
TP^e^									
No	345 (99.424)	1682 (99.763)	0.605	341 (99.42)	1446 (99.72)	0.323	14 (100)	320 (100)	NA
Yes	2 (0.576)	4 (0.237)		2 (0.58)	4 (0.28)		0 (0)	0 (0)	
Herpes virus^e^									
No	345 (99.424)	1685 (99.941)	0.077	341 (99.42)	1449 (99.93)	0.096	14 (100)	320 (100)	NA
Yes	2 (0.576)	1 (0.059)		2 (0.58)	1 (0.07)		0 (0)	0 (0)	
Mycoplasma genitalium^e^									
No	291 (80.387)	1504 (84.637)	0.045	274 (79.88)	1220 (84.14)	0.064	13 (92.86)	278 (86.88)	1.000
Yes	71 (19.613)	273 (15.363)		69 (20.12)	230 (15.86)		1 (7.14)	42 (13.13)	
Chlamydia genitalium^e^									
No	340 (93.923)	1716 (96.894)	0.006	321 (93.59)	1396 (96.61)	0.010	14 (100)	313 (98.12)	1.000
Yes	22 (6.077)	55 (3.106)		22 (6.41)	49 (3.39)		0 (0)	6 (1.88)	
Condyloma acuminata^e^									
No	343 (98.847)	1681 (99.703)	0.081	339 (98.83)	1446 (99.72)	0.048	14 (100)	319 (99.69)	NA
Yes	4 (1.153)	5 (0.297)		4 (1.17)	4 (0.28)		0 (0)	1 (0.31)	
HPV^e^									
No	341 (98.271)	1663 (98.636)	0.784	338 (98.54)	1433 (98.83)	0.593	14 (100)	314 (98.13)	1.000
Yes	6 (1.729)	23 (1.364)		5 (1.46)	17 (1.17)		0 (0)	6 (1.88)	

*PCOS* polycystic ovarian syndrome, *TP* treponema pallidum antibody, *HPV* high-risk human papillomavirus

^a^Faliure to conceive after regular unprotected sexual intercourse for 1 year

^b^The sum does not necessarily equal the sample size for all variables because of missing data

^c^Pearson’s χ^2^ test

^d^The diagnosis standard referred to the Dutch Rotterdam diagnostic criteria

^e^Make negative results as reference groups

Regarding male disease history (Table 3), prostatitis and lower genital tract infection (mycoplasma and chlamydia) were factors that were associated with infertility in the “first child intention” group. However, no significant differences were found in male disease history in the “second child intention” group between infertility and fertility.

**Table 3:** Medical history of men all included

	All couples		First child		P value^c^	Second child		P value^c^
	Fertility (N = 1808)	Infertility^a^ (N = 369)	Fertility (N = 1484)	Infertility^a^ (N = 355)		Fertility (N = 324)	Infertility^a^ (N = 14)	
	n^b^ (%)	n^b^(%)	n^b^ (%)	n^b^(%)		n^b^ (%)	n^b^(%)	
Genital tract infection history								
Neisseria gonorrhoeae								
No	1682 (99.94)	346 (100.00)	1362 (99.93)	327 (100.00)	1.000	313 (100.00)	14 (100.00)	NA
Yes	1 (0.06)	0	1 (0.07)	0 (0.00)		0 (0.00)	0 (0.00)	
TP								
No	1683 (100.00)	346 (100.00)	1363 (100.00)	327 (100.00)	NA	313 (100.00)	14 (100.00)	NA
Yes	0	0	0 (0.00)	0 (0.00)		0 (0.00)	0 (0.00)	
Herpes virus								
No	1681 (99.88)	346 (100.00)	1362 (99.93)	327 (100.00)	1.000	312 (99.68)	14 (100.00)	1.000
Yes	2 (0.12)	0	1 (0.07)	0 (0.00)		1 (0.32)	0 (0.00)	
Mycoplasma genitalium								
No	1648 (97.92)	328 (94.80)	1331 (97.65)	310 (94.80)	0.009	310 (99.04)	13 (92.86)	0.161
Yes	35 (2.08)	18 (5.20)	32 (2.35)	17 (5.20)		3 (0.96)	1 (7.14)	
Chlamydia genitalium								
No	1658 (98.51)	267 (96.04)	1340 (98.31)	314 (96.02)	0.017	311 (99.36)	14 (100.00)	1.000
Yes	25 (1.49)	13 (3.76)	23 (1.69)	13 (3.98)		2 (0.64)	0 (0.00)	
HPV								
No	1682 (99.94)	345 (99.71)	1362 (99.93)	326 (99.69)	0.350	313 (100.00)	14 (100.00)	NA
Yes	1 (0.06)	1 (0.29)	1 (0.07)	1 (0.31)		0 (0.00)	0 (0.00)	
Prostatitis								
No	1627 (97.66)	321 (94.69)	1312 (97.35)	304 (95.00)	0.050	308 (99.35)	13 (92.86)	0.124
Yes	39 (2.34)	18 (5.31)	37 (2.74)	16 (5.00)		2 (0.65)	1 (7.14)	

*TP* treponema pallidum antibody, *HPV* high-risk human papillomavirus

^a^Faliure to conceive after regular unprotected sexual intercourse for 1 year

^b^The sum does not necessarily equal the sample size for all variables because of missing data

^c^Pearson’s χ^2^ test

### Multivariate analysis of risk factors for infertility

Multicollinearity diagnosis was performed before multivariate analysis, and there was no significant collinearity among the factors included in the multivariate analysis (Additional file 1: Table S1). Table 4 displays the multivariate analysis of infertility risk. For couples intending to have their first child, high BMI (≥ 24 kg/m^2^) and older age (> 35 years) for women and low BMI (< 18.5 kg/m^2^) for men were risk factors for infertility. As for female menstrual history, our results indicated that women with longer menstrual durations (OR = 4.47, 95% CI 2.25–8.88) were at a greater risk for infertility. Moreover, women with a history of endometrial polyps (OR = 2.52, 95% CI 1.28–4.97), PCOS (OR = 6.72, 95% CI 1.79–7.39), endometriosis (OR = 2.52, 95% CI 1.27–4.97), or mycoplasma infection in the lower genital tract (OR = 1.54, 95% CI 1.09–2.40) were more likely to experience infertility than women who did not. Additionally, previous salpingostomy (OR = 3.44, 95% CI 1.68–7.07) was also associated with a higher risk of infertility.

**Table 4:** Multivariable logistic regression analysis predicting risk factors for infertility

	All couples		First child		Second child	
	AOR [95% CI]	P value	AOR [95% CI]	P value	AOR [95% CI]	P value
Age, years, women						
20–24	Reference	0.008	Reference	< 0.001	Reference	0.040
25–29	1.41 [0.64, 3.09]		1.23 [0.67, 2.12]		1.02 [0.57, 1.86]	
30–34	1.96 [0.89, 4.32]		1.54 [0.87, 3.01]		0.97 [0.45, 1.34]	
35–39	2.09 [0.88, 4.96]		1.70 [1.27, 2.28]		0.55 [0.13, 2.46]	
≥ 40	5.93 [1.79, 19.67]		7.89 [2.41, 25.79]		7.36 [1.01, 53.84]	
BMI, kg/m^2^, women						
< 18.5	1.20 [0.78, 1.84]	0.017	1.06 [0.68, 1.64]	0.017	1.46 [0.14, 15.70]	0.894
18.5–23.9	Reference		Reference		Reference	
24–27.9	1.39 [0.90, 2.13]		1.58 [1.01, 2.45]		0.52 [0.05, 5.82]	
≥ 28	2.73 [1.40, 5.32]		2.86 [1.31, 6.26]		NA	
BMI, kg/m^2^, men						
< 18.5	3.41 [1.51, 7.71]	0.033	3.09 [1.42, 6.74]	0.017	NA	0.965
18.5–23.9	Reference		Reference		Reference	
24–27.9	1.11 [0.81, 1.52]		1.37 [0.99, 1.90]		1.47 [0.35, 6.23]	
≥ 28	1.07 [0.70, 1.65]		1.26 [0.82, 1.94]		NA	
Menstrual duration, days						
< 2	NA	0.035	NA	< 0.001	NA	
2–7	Reference		Reference		Reference	0.998
> 7	2.34 [1.23, 4.45]		4.47 [2.25, 8.88]		0.99 [0.10, 9.89]	
Leiomyoma						
No	Reference	0.013	Reference	0.119	Reference	0.043
Yes	1.66 [1.11, 2.46]		1.38 [0.92, 2.07]		5.60 [1.06, 29.76]	
Endometrial polyp						
No	Reference	< 0.001	Reference	< 0.001	Reference	0.578
Yes	2.87 [1.67, 4.94]		2.52 [1.28, 4.97]		1.99 [0.18, 22.32]	
PCOS						
No	Reference	< 0.001	Reference	< 0.001	NA	–
Yes	3.89 [2.28, 6.64]		6.72 [1.79,7.39]		NA	
Endometriosis						
No	Reference	< 0.001	Reference	0.001	NA	–
Yes	3.65 [1.92, 6.95]		2.52 [1.27, 4.97]		NA	
Salpingostomy						
No	Reference	< 0.001	Reference	< 0.001	Reference	0.130
Yes	4.04 [2.04, 7.98]		3.44 [1.68, 7.07]		9.28 [0.52, 166.22]	
Chlamydia genitalium, women						
No	Reference	0.024	Reference	0.015	Reference	0.587
Yes	1.96 [1.09, 3.52]		1.54 [1.09, 2.40]		0.53 [0.05, 5.20]	

*BMI* body mass index, *PCOS* polycystic ovarian syndrome, *AOR* adjusted odds ratio, *CI* confidence interval

Nagelkerke R2 = 0.622

Regarding the “second-child intention” group, the ORs were statistically significant when females aged over 40 (OR = 7.36, 95% CI 1.01–53.84), while the female history of leiomyoma (OR = 5.60, 95% CI 1.06–29.76) was a significant risk factor for infertility in the “couples with second child intention” group, and was not significantly different in the “couples with first child intention” group (OR = 1.38, 95% CI = 0.92–2.07).

## Discussion

The overall incidence of infertility was 16.95% in Shanghai, which is much higher than the infertility rate reported in Shanghai 15 years ago. Our study found the incidence of “first child intention” and “second child intention” infertility as 19.30% and 4.14%, respectively. To our knowledge, this is the first study analyzing infertility incidence and risk factors in couples intending to have a first and second child. As seen from the above infertility rate, the infertility incidence of “second child intention” is significantly lower than that of “first child intention.” Our study also found differences in infertility risk factors between the two groups. For couples with “first child intention,” obesity (BMI ≥ 24 kg/m^2^), advanced age (> 30 years old), female gynecological diseases such as endometrial polyps, PCOS, endometriosis, mycoplasma infection of the lower reproductive tract, and previous surgical history of tubal infertility were all associated with infertility. For couples with “second child intention,” only the following variables were significantly related to infertility: age over 40 and leiomyoma.

Age is one of the causes of infertility [21]. In our study, women intending to have a first child, aged 35–39, were associated with a higher risk of infertility. In the “second child intention” group, couples 40 years and older were significantly associated with infertility. There could be three major reasons for this: (1) women with “second child intention” experienced a successful pregnancy, which suggests that these couples have a complete chain from the production of eggs and sperm to the success of pregnancy; (2) older women are less likely to develop new ovulatory dysfunction [22]. Based on the rapid decline in fertility of women after age 40 [23], compared with women aged 20–24, women over the age of 40 had a significantly increased risk of infertility in both the “first child intention” and “second child intention” groups. Due to physiological processes such as ovarian reserve and decreased sperm quality, fertility rapidly declines in this age group, which greatly increases the incidence of infertility [23, 24].

The incidence of infertility in Shanghai is currently almost double what it was in 2002 and exceeds the incidence of infertility in China [20]. This could be related to Shanghai's social, cultural, and economic development in the past 15 years [5]. Economic development has brought tremendous social progress and has caused people to pursue education, careers, and higher incomes, resulting in couples that do not become pregnant in early adulthood. Previous studies have suggested that it is best for women to become pregnant before they are 35 [25]. As females age, ovarian function declines, as does fertility [26]. Work stress and occupational exposure also affect women's endocrine function, which endangers fertility [27]. 95.27% of the women in this study had stable jobs. Due to increasing work pressure and hours worked by women in recent years, the resulting exhaustion and mental stress could promote the secretion of an adrenocortical hormone-releasing hormone. This interrupts the normal gonadal feedback in the brain, thereby affecting a series of conception processes and reducing the clinical pregnancy rate [28].

Similar to studies on infertility rates in other regions of China [9, 20], this study found that the annual infertility rate from 2013 to 2017 increased, especially after 2016, and ultimately reached 21.4%. This is most likely related to 2016 changes in the national population policy when the universal two-child policy was implemented, increasing the proportion of elderly and high-risk pregnant women [29]. Additionally, as mentioned above, the increasing work intensity in recent years has also increased infertility rates.

Numerous studies have shown the effects of the female reproductive history and gynecological history on infertility [30, 31]. Our investigation confirmed that factors related to infertility for women included menstrual history (long menstrual cycle or long menstrual duration), gynecological history (leiomyoma, PCOS, endometrial polyps, or endometriosis), surgical history (salpingostomy), and infection history (chlamydia). Additionally, several researchers have explained the relationship between these risk factors and infertility, which could be related to ovulation disorders, deterioration of the intrauterine environment, and pathological changes in the cervix [32, 33].

The prevalence of infertility can be altered using various approaches. Primary infertility is commonly calculated by the DHS-type (Demographic and Health Survey) infertility measure [34], while some scholars have applied the novel current duration approach and calculated that the prevalence of infertility was two times higher than the traditionally constructed measure [35]. These studies emphasize that the definition and methodological methods are important when estimating the prevalence of infertility. Our study established a prospective cohort to obtain infertility incidence rates, focusing on the frequency of new infertility in a subset of the population while reducing recall bias. On the other hand, prevalence was assessed using cross-sectional studies that analyze the ratio of new and old disease cases in a population over a period of time. Therefore, the incidence of infertility and related risk factors obtained in this study have clinical importance.

We conducted the first studies assessing the incidence and risk factors of infertility in couples seeking to have a first and second child. However, our study had some limitations. First, because male infertility factors were not fully covered, we did not identify any significant risk factors related to male infertility. Second, as the prospective cohort was established from a single institution, this study did not use the Probability Proportional to Size (PPS) sampling method; simple random sampling was used to recruit research objects. As proportional sampling methods, simple random sampling can ensure equal opportunities for each subject in the target population and effectively obtain representative samples in this study [36]. Although our sample was obtained from the community and the sampling method was effective, our cohort was based on a cohort established by pre-pregnancy centers, meaning there was some bias in how the overall population was represented. Finally, based on our analysis of infertility incidence rather than infertility prevalence, the study excluded infertile people at the inclusion stage. This could have produced differing research results, and particularly underestimated factors such as some gynecological diseases that affect infertility. Therefore, future research design should include more samples and an infertile population.

## Conclusions

This survey showed that the infertility rate is approximately 16.95% in our sample. The incidence of infertility in the “first child intention” and “second child intention” groups was 19.30% and 4.14%, respectively. Our data also showed risk factors related to infertility in the “first child intention” and “second child intention” groups. However, some mechanisms leading to infertility are still unclear and require further study. To some extent, these results can inform government and medical institutions seeking to make policies governing population size. Age is an important risk factor for infertility, and couples who marry when they are older is an important factor affecting overall fertility. Medical workers can use their influence to inform couples of the benefits and drawbacks of having children at certain ages, while the government can strengthen fertility subsidies to couples of childbearing ages to increase fertility levels.

## Supplementary Information

**Additional file 1: Table S1.** Multi-collinearity analysis between independent variables included in the regression models.

## Data Availability

All data generated or analyzed during this study are included in this published article.

## Abbreviations

ORs: Odds ratios
CIs: Confidence intervals
BMI: Abnormal body mass index
PCOS: Polycystic ovarian syndrome
ART: Assisted reproductive technology
WHO: World Health Organization
DHS-type: Demographic and Health Survey
SD: Standard deviation
IVF-ET: In vitro fertilization and embryo transfer
ICSI: Intracytoplasmic sperm injection
PGD: Preimplantation genetic diagnosis
PPS: Probability Proportional to Size
